# Supplementary material for: VGLL3 is a mechanosensitive protein that promotes cardiac fibrosis through liquid–liquid phase separation
Source: Nat Commun. 2023 Feb 8;14:550. doi: 10.1038/s41467-023-36189-6 (PMC9908974; doi:10.1038/s41467-023-36189-6)

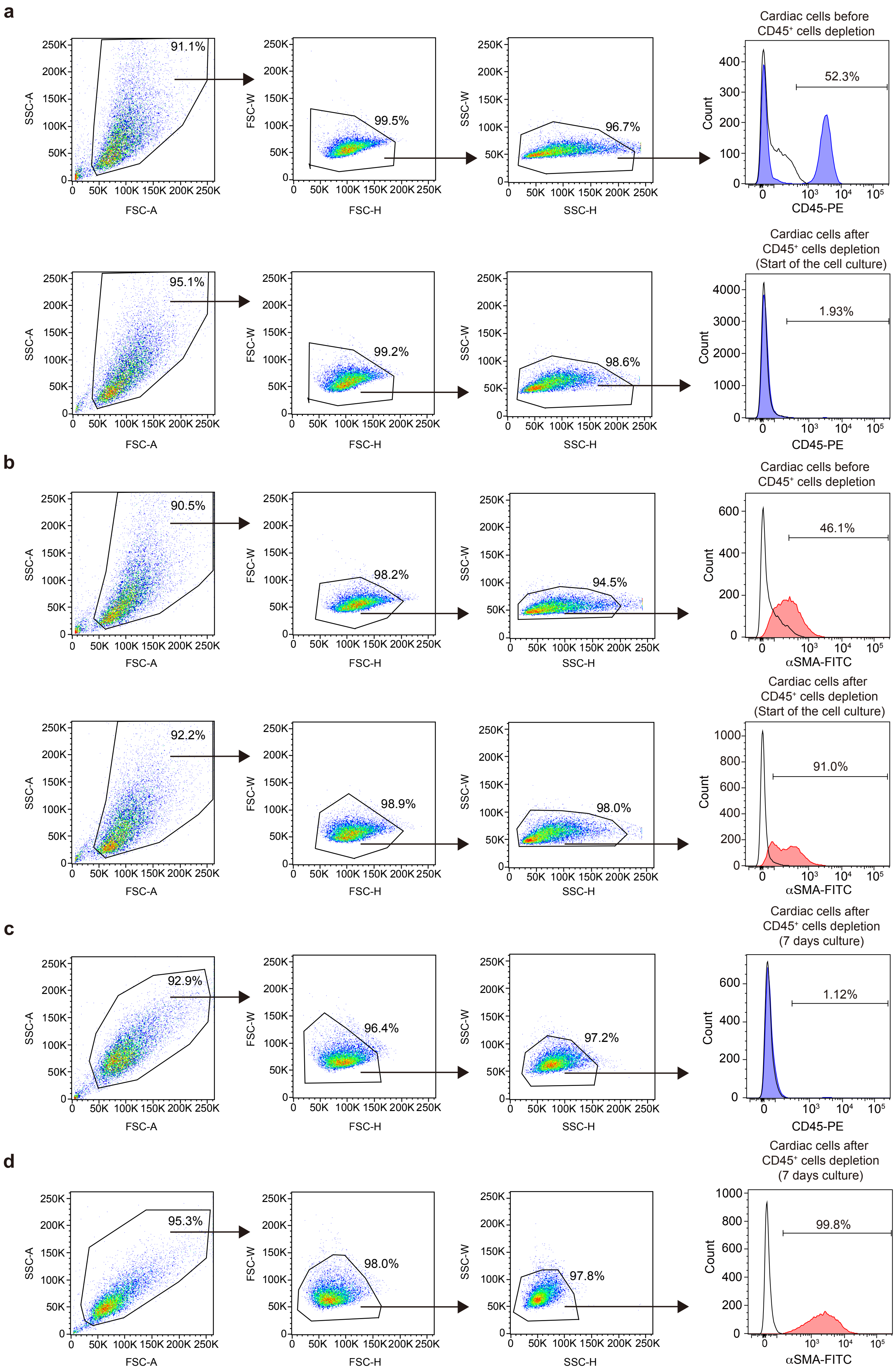
a
Cardiac cells before CD45⁺ cells depletion
91.1%
99.5%
96.7%
52.3%
Cardiac cells after CD45⁺ cells depletion (Start of the cell culture)
95.1%
99.2%
98.6%
1.93%
b
Cardiac cells before CD45⁺ cells depletion
90.5%
98.2%
94.5%
46.1%
Cardiac cells after CD45⁺ cells depletion (Start of the cell culture)
92.2%
98.9%
98.0%
91.0%
c
Cardiac cells after CD45⁺ cells depletion (7 days culture)
92.9%
96.4%
97.2%
1.12%
d
Cardiac cells after CD45⁺ cells depletion (7 days culture)
95.3%
98.0%
97.8%
99.8%
SSC-A
FSC-A
FSC-W
FSC-H
SSC-W
SSC-H
Count
CD45-PE
αSMA-FITC

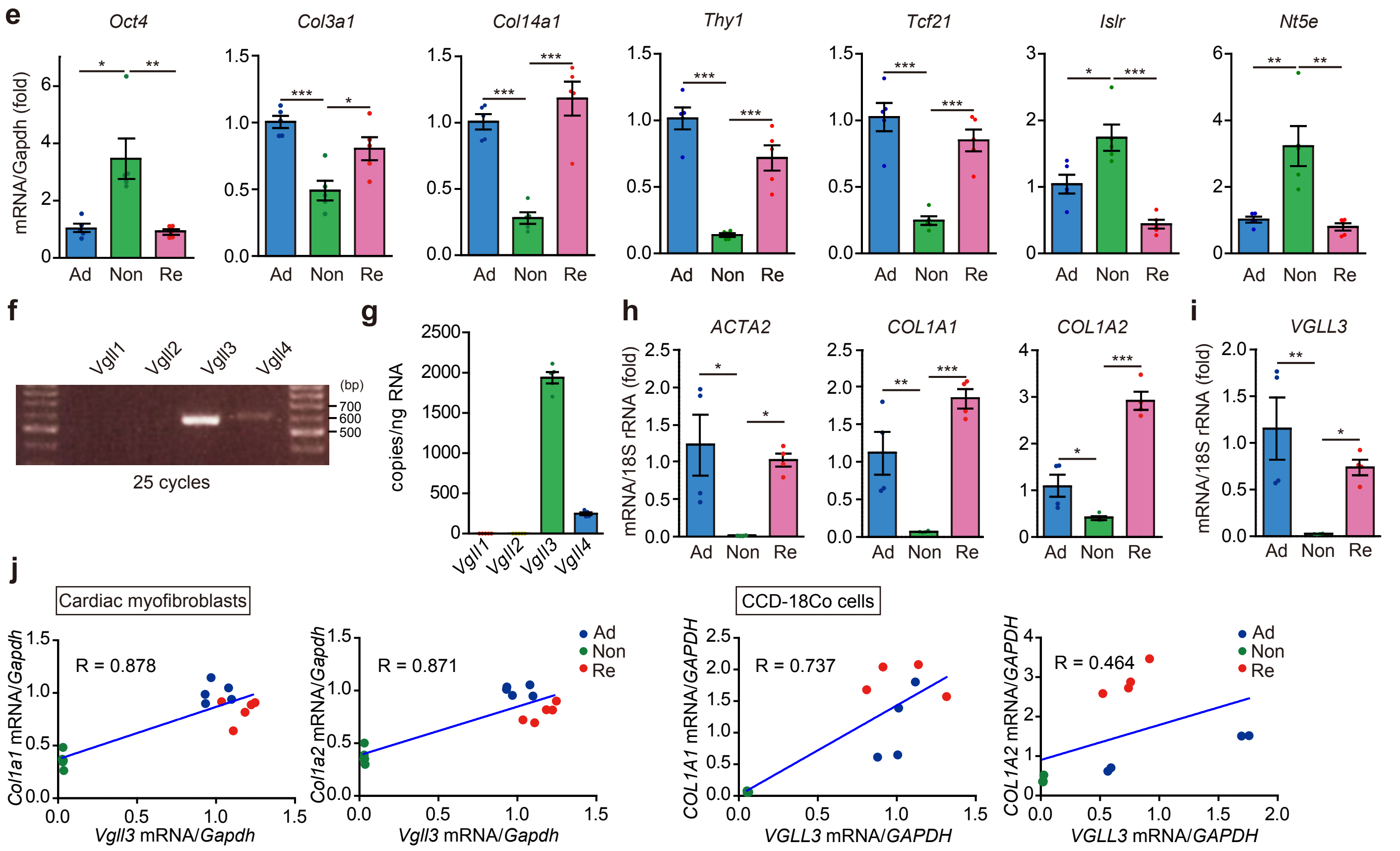

Supplementary Fig. 1 Purification of cardiac myofibroblasts and mRNA expression of stem cell marker, fibroblast markers, mesenchymal stem cell markers, and *Vgll* family members in cardiac myofibroblasts.

a-**d** Flow cytometry profiles of cardiac cells isolated from the myocardial infarction (MI) model mice on day 3 before and after CD45-positive cell depletion using magnetic-activated cell sorting (MACS). Before depletion, the cardiac cell fraction contained 52.3% of CD45-positive hematopoietic cells (**a,** upper pannels) and 46.1% of αSMA-positive myofibroblasts (**b**, upper pannels). After CD45-positive cell depletion by MACS, few CD45-positive hematopoietic cells were detected in the cardiac cell fraction (**a,** lower pannels), and almost all cells were αSMA-positive myofibroblasts (91.0%) (**b**, lower pannels). When the cardiac cell fraction after removal of CD45-positive cells was cultured for 7 days, CD45-positive hematopoietic cells were hardly detected in the cell fraction (**c**), which contained αSMA-positive myofibroblasts with high purity (99.8%) (**d**). Histograms filled with blue or red represent cardiac cells stained with PE-conjugated anti-CD45 antibody or FITC-conjugated anti-αSMA antibody, respectively. Unfilled histograms represent the unstained control. **e** *Oct4*, *Col3a1*, *Col14a1*, *Thy1*, *Tcf21*, *Islr*, and *Nt5e* mRNA levels in Adherent (Ad), Non-adherent (Non), and Re-adherent (Re) cardiac myofibroblasts (n = 5 each). **f** PCR analysis for the mRNA expression of VGLL family (*Vgll1*, *Vgll2*, *Vgll3*, and *Vgll4*) in cardiac myofibroblasts. **g** Absolute quantification of *Vgll1*, *Vgll2*, *Vgll3*, and *Vgll4* expression in cardiac myofibroblasts by qRT-PCR (n = 5 each). **h**,**i** *ACTA2*, *COL1A1*, *COL1A2*, and *VGLL3* mRNA levels in Ad, Non, and Re CCD-18Co cells (n = 4 each). **j** Scatter plots for the correlation between *Vgll3* mRNA expression and expression of *Col1a1* and *Col1a2* in cardiac myofibroblasts or CCD-18Co cells cultured in Ad, Non, and Re. R represents the correlation coefficient. All the experiments were conducted at least three times. Data in **e**, **g**, **h**,and **i** are shown as the mean ± SEM. P-values were determined using the Kruskal–Wallis followed by Dunn's test in **e** (*Oct4*), one-way ANOVA followed by Tukey's range test in **e** (*Col3a1*, *Col14a1*, *Thy1*, *Tcf21*, *Islr*, and *Nt5e*), **h**, and **i**, $^{*}P < 0.05$, $^{**}P < 0.01$, $^{***}P < 0.001$.

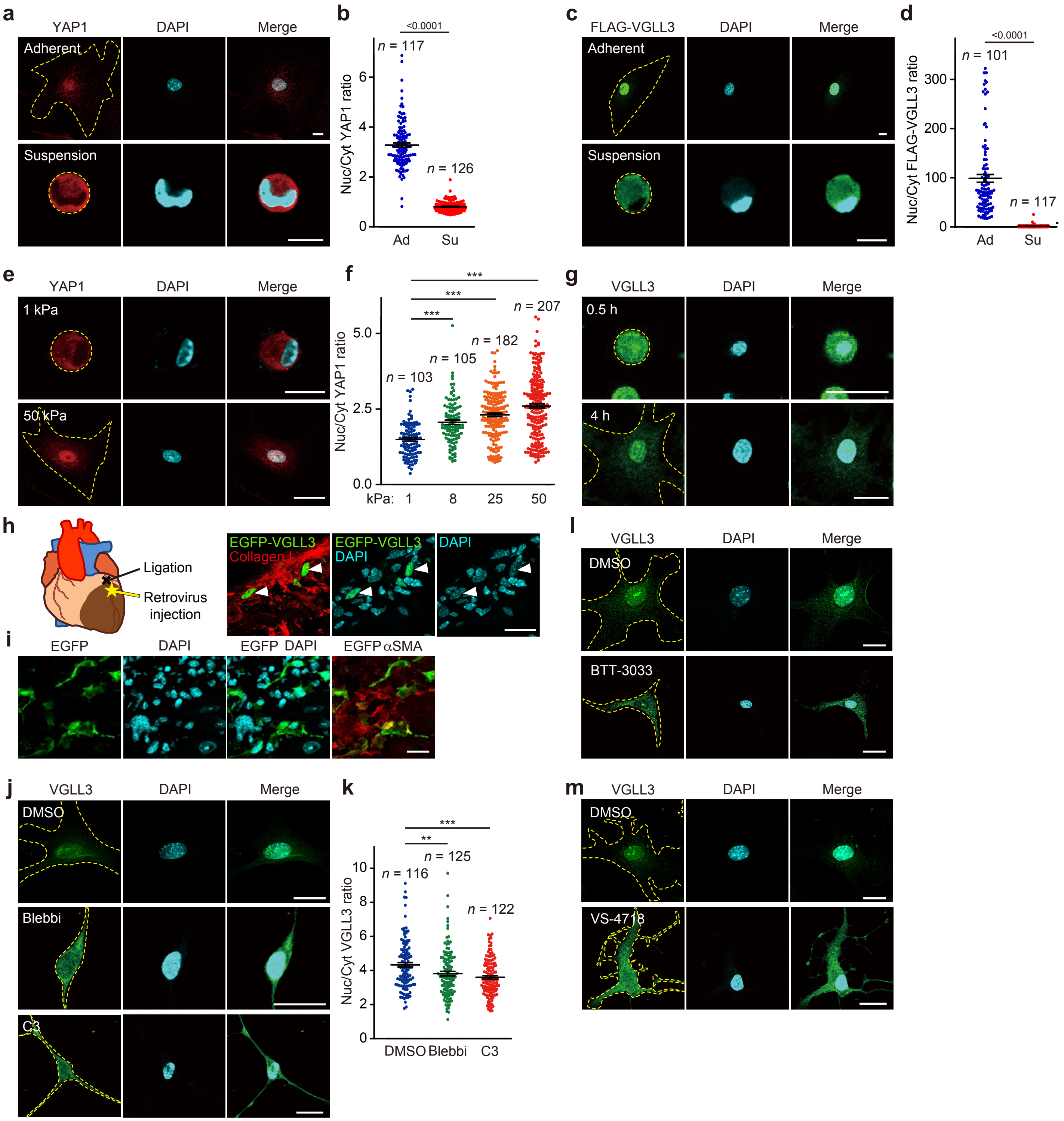

Supplementary Fig. 2 Nuclear translocation of YAP1 induced by substrate stiffness in cardiac myofibroblasts and the effects of blebbistatin and C3 transferase on VGLL3 nuclear translocation.

a Micrographs of myofibroblasts stained with anti-YAP1 antibody in adherent or suspension culture. **b** Ratios of nuclear to cytoplasmic YAP1 intensities in myofibroblasts in adherent (Ad) culture or in suspension (Su) culture. **c** Micrographs of myofibroblasts overexpressing FLAG-VGLL3 in adherent or suspension culture. **d** Ratios of nuclear to cytoplasmic FLAG-VGLL3 intensities in myofibroblasts in Ad culture or in Su culture. **e** Micrographs of myofibroblasts stained with anti-YAP1 antibody plated on soft (1 kPa) and stiff (50 kPa) hydrogels. **f** Ratios of nuclear to cytoplasmic YAP1 intensities in myofibroblasts plated on hydrogels with different stiffness. **g** Micrographs of myofibroblasts stained with anti-VGLL3 antibody adhered for the indicated times to glass-bottom dishes coated with poly-L-lysine. **h**,**i** Schematic representation of the site of intramyocardial injection. The retrovirus encoding EGFP or EGFP-VGLL3 was administered directly below the ligation site (left image of **h**). Nuclear localization of EGFP-VGLL3 in cardiac myofibroblasts around Collagen I deposition in the left ventricle of mouse hearts 3 days after MI (Right micrographs of **h**). White arrowheads in merged images indicated representative signals for EGFP-VGLL3 located in nuclei of myofibroblasts. In contrast, EGFP alone was distributed in cytoplasm of cardiac myofibroblasts (**i**). **j** Micrographs of myofibroblasts treated with DMSO (0.5%), blebbistatin (Blebbi, 50 μM), and C3 transferase (C3, 3 μg/mL). **k** Ratios of nuclear to cytoplasmic VGLL3 intensities in myofibroblasts treated with the cytoskeletal inhibitors as described in **j**. **l** Micrographs of myofibroblasts treated with DMSO (0.5%) and BTT-3033 (30 μM). **m** Micrographs of myofibroblasts treated with DMSO (0.5%) and VS-4718 (50 μM). Data are shown as the mean ± SEM. *P*-values were determined using the two-sided Mann–Whitney's *U* test in **b** and **d**, Kruskal-Wallis followed by Dunn's test in **f** and **k**, ****P* < 0.001. Confocal micrographs of YAP1, FLAG-VGLL3, VGLL3 and nuclei in cardiac myofibroblasts are representative of at least three biological replicates. Scale bars in **a**, **c**, **e**, **g**, **h**-**j**, **l** and **m** = 20 μm.

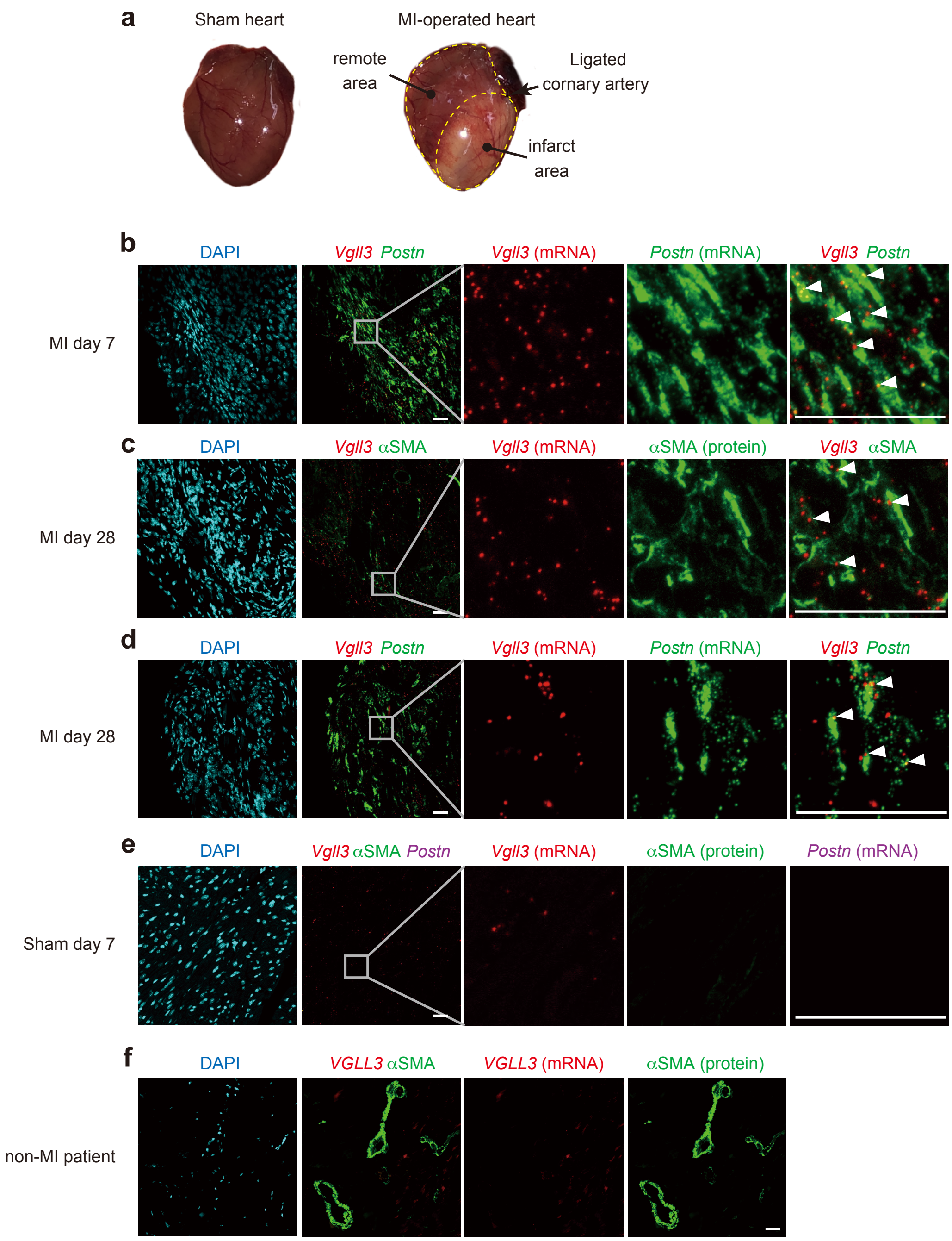

Supplementary Fig. 3 *Vgll3* is specifically expressed in myofibroblasts from fibrotic hearts.

a Determination of the infarct or remote area in a mouse heart, 3 days after MI. **b**–**f** Representative images of in situ hybridisation of *Vgll3* mRNA in the left ventricle of MI murine heart on day 7 (**b**), day 28 (**c**, **d**), sham murine heart on day 7 (**e**) and the heart of a non-MI patient (**f**). Co-in situ hybridisation of *Vgll3* and *Postn* was performed on heart sections (**b**, **d**, **e**). Heart sections were co-stained with αSMA (**c**, **e**, **f**). All experiments were conducted at least three times. Scale bars in **b**–**f** = 30 μm. Grey squares in **b**–**e** indicate areas magnified in the right panels. White arrowheads in merged images (**b**–**d**) indicate the representative signals for *Vgll3* mRNA in myofibroblasts.

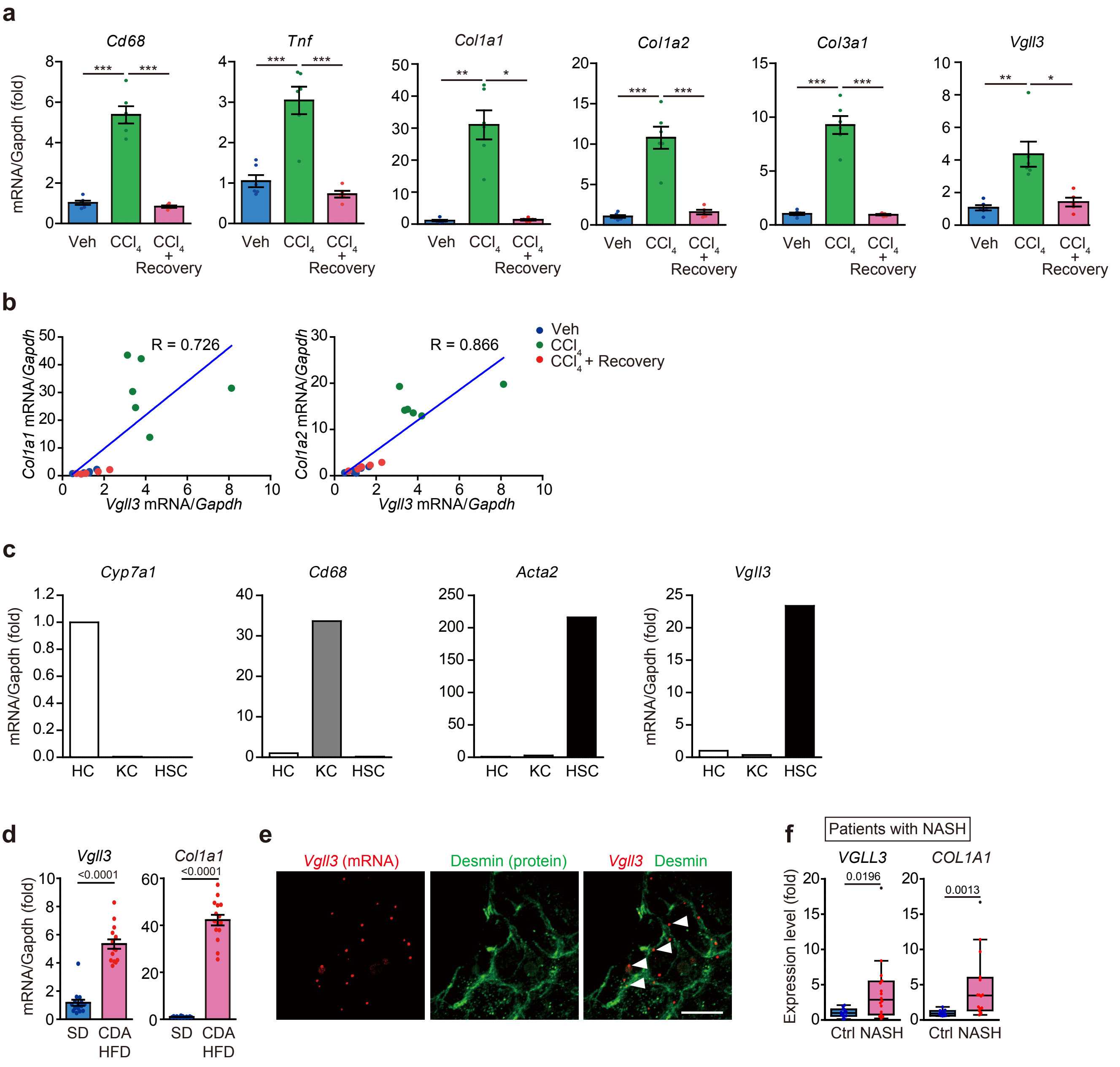

Supplementary Fig. 4 *Vgll3* is induced in fibrotic liver and is specifically expressed in hepatic stellate cells.
a *Cd68*, *Tnf*, *Col1a1*, *Col1a2*, *Col3a1*, and *Vgll3* mRNA levels in mouse liver after administration of the vehicle (Veh), CCl_4, or CCl_4 followed by recovery for 4 weeks (CCl_4+Recovery) (n = 6, 6, and 5, respectively). **b** Scatter plots for the correlation between *Vgll3* mRNA expression and expression of *Col1a1* and *Col1a2* in mouse livers (Veh, CCl_4, or CCl_4+Recovery). R represents the correlation coefficient. **c** mRNA levels of the hepatocyte marker (*Cyp7a1*), the Kupffer cell marker (*Cd68*), and the hepatic stellate cell marker (*Acta2*) in cell fractions of hepatocytes (HC), Kupffer cells (KC), and hepatic stellate cells (HSC) isolated from the liver of a CCl_4-administered mouse. **d** mRNA expression of *Vgll3* in HC, KC, and HSC. Results are expressed as fold changes relative to Veh (**a**) or HC (**c**) data. **d** *Vgll3* and *Col1a1* mRNA levels in the liver of mice fed a standard diet (SD) or choline-deficient, L-amino acid-defined, high-fat diet (CDAHFD) (n = 15 each). **e** Co-detection of *Vgll3* mRNA and desmin in the liver of non-alcoholic steatohepatitis (NASH) model mice. **f** *VGLL3* and *COL1A1* mRNA levels in the livers of control individuals (n = 14) and patients with NASH (n = 16) based on data from GSE126848. Data in **f** are presented as box and whisker plots (Tukey style, outliers in black dots). The box shows the 25th to 75th percentile range with the median value represented by a horizontal line. The whiskers stretch to the minimum and maximum values within 1.5 times the interquartile range from the 25th–75th percentiles. Data in **a**, **d** and **f** are presented as the mean ± SEM. *P*-values were determined using one-way ANOVA followed by Tukey's range test in **a** (*Cd68*, *Tnf*, *Col1a2*, and *Col3a1*),the Kruskal–Wallis test followed by Dunn's test in **a** (*Col1a1* and *Vgll3*), the two-sided Student's *t*-test in **d**, and the two-sided Mann–Whitney's *U* test in **f**. $^*P < 0.05$, $^{**}P < 0.01$, $^{***}P < 0.001$. *In situ* hybridisation data are representative of at least three independent experiments. White arrowheads in **e** indicate the representative signals for *Vgll3* mRNA. Scale bar in **e** = 10 μm.

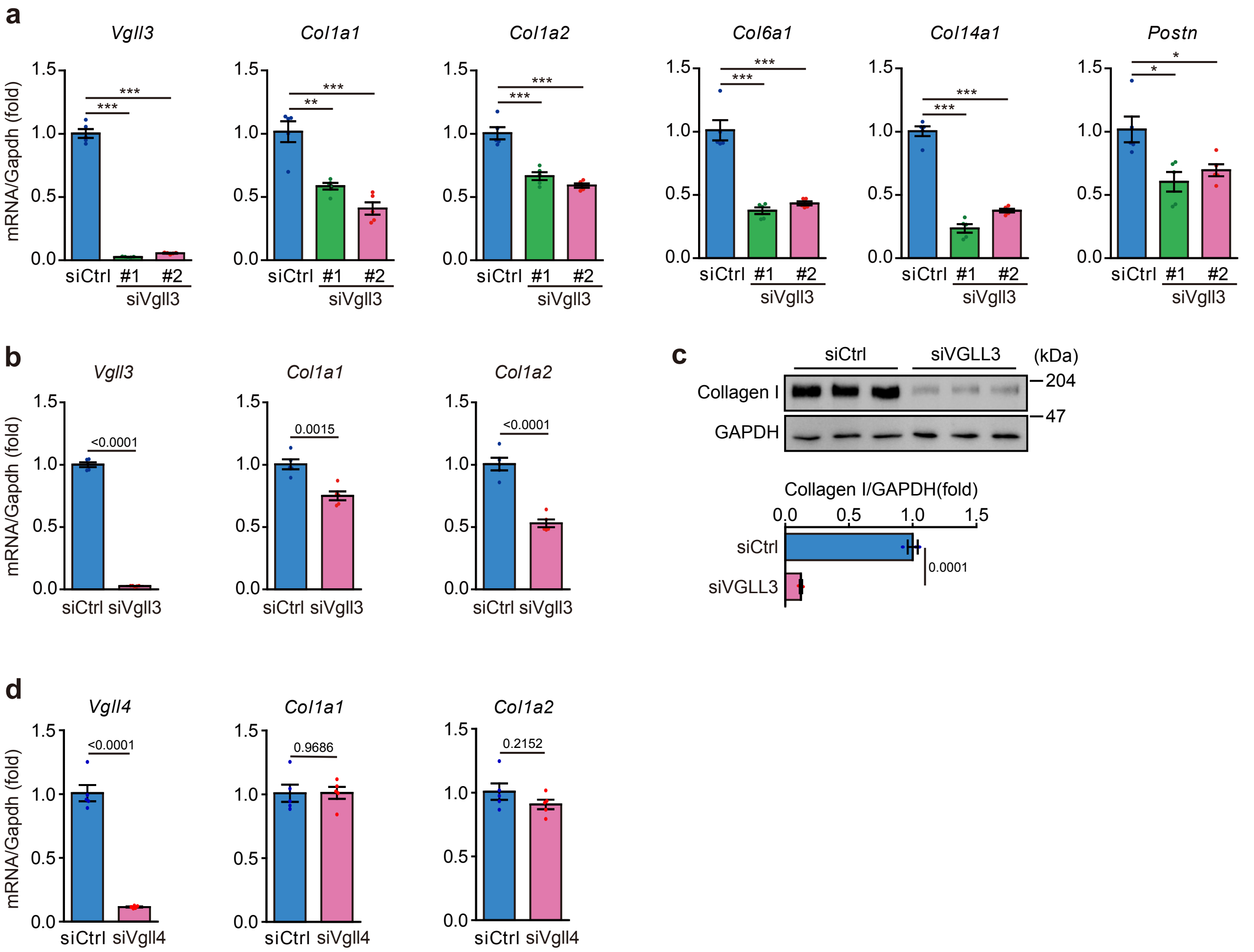

Supplementary Fig. 5 Transfection with siRNA against *Vgll3* suppressed the expression of fibrosis-related genes in cardiac myofibroblasts and hepatic stellate cells.

a mRNA levels of *Vgll3* and fibrosis-related genes in cardiac myofibroblasts transfected with different siRNAs targeting *Vgll3* (si*Vgll3* #1 and #2) (*n* = 5 each). **b** mRNA levels of *Vgll3* and fibrosis-related genes in hepatic stellate cells isolated from CCl_4-induced fibrotic mouse livers, transfected with siRNA (#1) targeting *Vgll3* (si*Vgll3*) (*n* = 5 each). **c** Protein levels of Collagen I and GAPDH in CCD-18Co transfected with siRNA (#1) targeting *VGLL3* (*n* = 3 each). **d** mRNA levels of *Vgll4* and fibrosis-related genes in cardiac myofibroblasts transfected with siRNA targeting *Vgll4* (*n* = 5 each). All the experiments were performed at least three times. Data are presented as the mean ± SEM. *P*-values were determined using one-way ANOVA followed by Tukey's range test in **a**, and two-sided Student's *t*-test in **b**-**d**, *$P < 0.05$, **$P < 0.01$, ***$P < 0.001$. n.s.; not statistically significant.

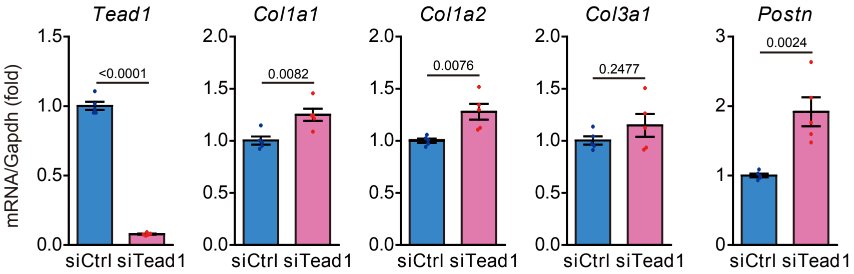

Supplementary Fig. 6 Transfection with siRNA against *Tead1* did not suppress the expression of fibrosis-related genes in cardiac myofibroblasts.

mRNA levels of fibrosis-related genes in cardiac myofibroblasts transfected with siRNA targeting *Tead1* (*n* = 5 each). The experiments were performed at least three times. Data are shown as the mean ± SEM. *P*-values were determined using the two-sided Student's *t*-test. n.s.; not statistically significant.

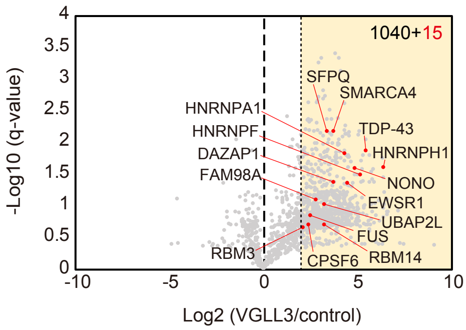

Supplementary Fig. 7 Identification of fifteen paraspeckle proteins as VGLL3 interactors by mass spectrometry. Fifteen paraspeckle proteins are indicated in the volcano plot of Fig. 4a.

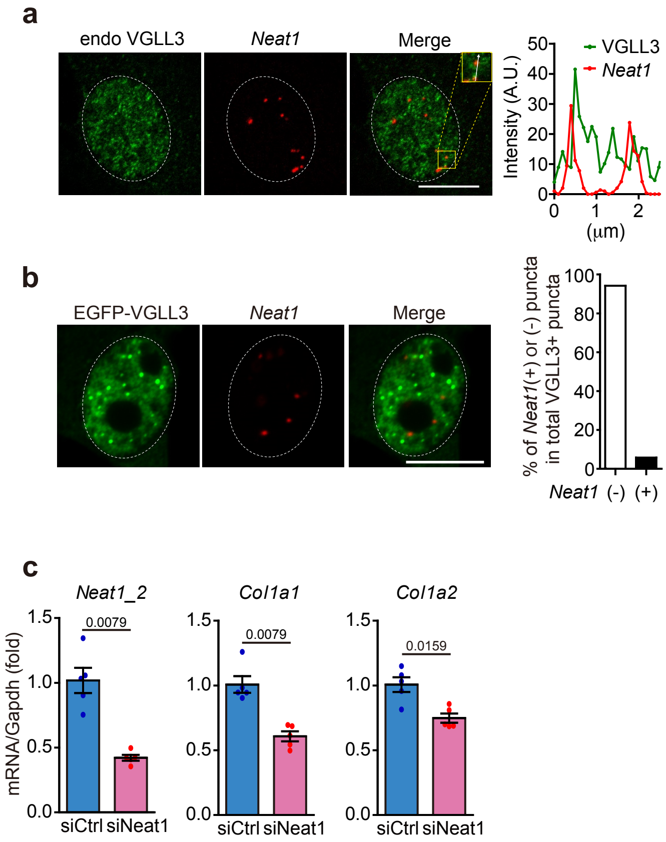

Supplementary Fig. 8 Localisation of *Neat1* in cardiac myofibroblasts and its contribution to their collagen production. **a** Micrographs of endogenous VGLL3 puncta detected by immunostaining, and *Neat1* mRNA detected by RNA-FISH in cardiac myofibroblasts. The graph represents line scans along the white line in the yellow dashed square in the inset. **b** Micrographs of EGFP-VGLL3 puncta and *Neat1* mRNA detected by RNA-FISH in NIH3T3 cells. Percentage of *Neat1*-postive or *Neat1*-negative VGLL3 puncta in all VGLL3 puncta (n = 133 puncta) is shown in the graph. **c** mRNA levels of *Neat1_2* (long isoform of *Neat1*, essential for the assembly of paraspeckles), *Col1a1*, and *Col1a2* in cardiac myofibroblasts transfected with siRNA targeting *Neat1* (n = 5 each). The experiments were performed at least three times. Data in **c** are shown as the mean ± SEM. *P*-values were determined using the two-sided Mann–Whitney's *U* test in **c**. Scale bars in **a** and **b** = 10 µm.

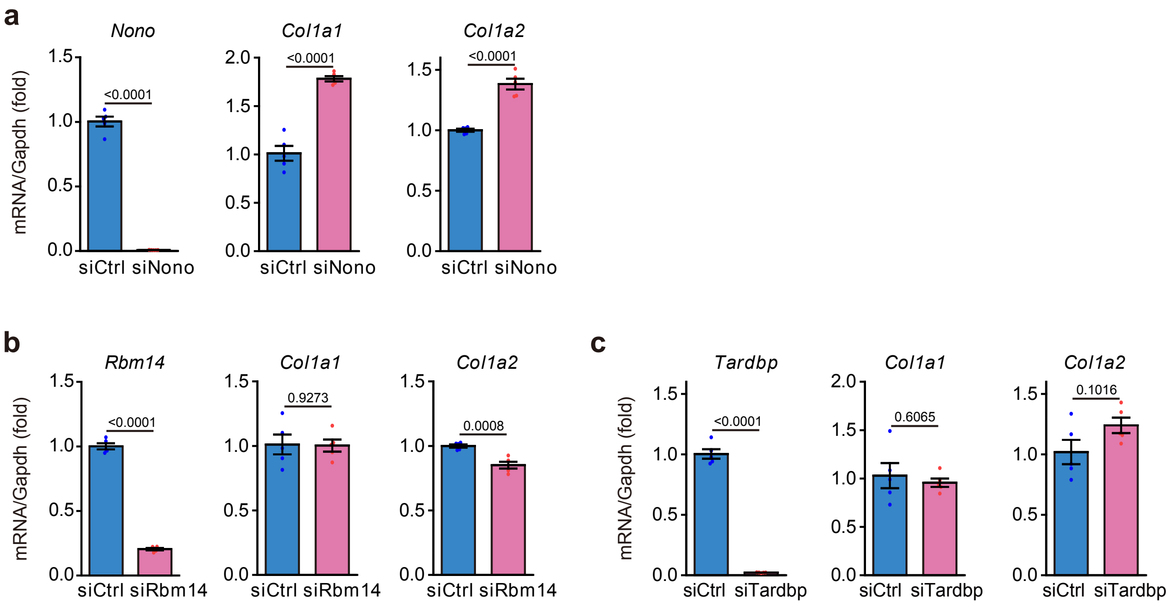

Supplementary Fig. 9 The effect of paraspeckle proteins identified as VGLL3 interactors on collagen production by cardiac myofibroblasts.

a-**c** mRNA levels of collagens in cardiac myofibroblasts transfected with siRNA targeting *Nono* (**a**), *Rbm14* (**b**) or *Tardbp* (**c**) (*n* = 5 each). The experiments were performed at least three times. Data are shown as the mean ± SEM. *P*-values were determined using the two-sided Student's *t*-test.

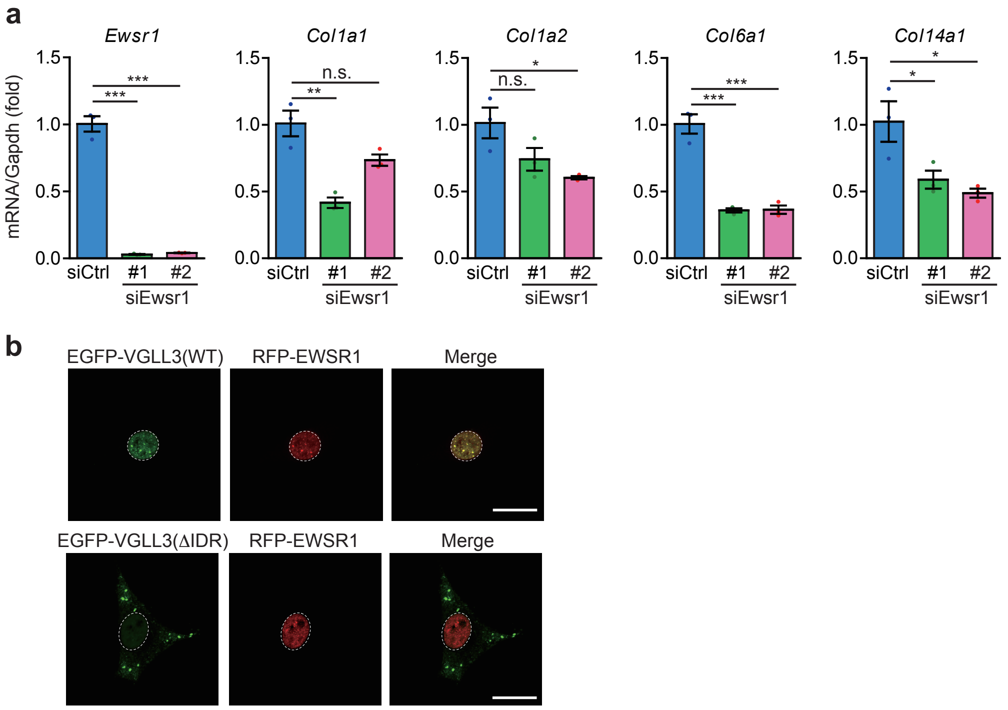

Supplementary Fig. 10 EWSR1 promotes collagen expression.

a mRNA levels of *Ewsr1* and collagens in cardiac myofibroblasts transfected with different siRNAs targeting *Ewsr1* (siEwsr1 #1 and #2) (n = 3 each). Data are shown as the mean ± SEM. **b** Images of live NIH3T3 cells transfected with EGFP-VGLL3 (WT) or (ΔIDR) and RFP-EWSR1. The experiments were done three times. P-values were determined using one-way ANOVA followed by Tukey's range test in **a**, $^{*}P < 0.05$, $^{**}P < 0.01$, $^{***}P < 0.001$. n.s.; not statistically significant. White dashed circles mark the nucleus in **b**. Scale bars in **b** = 20 μm.

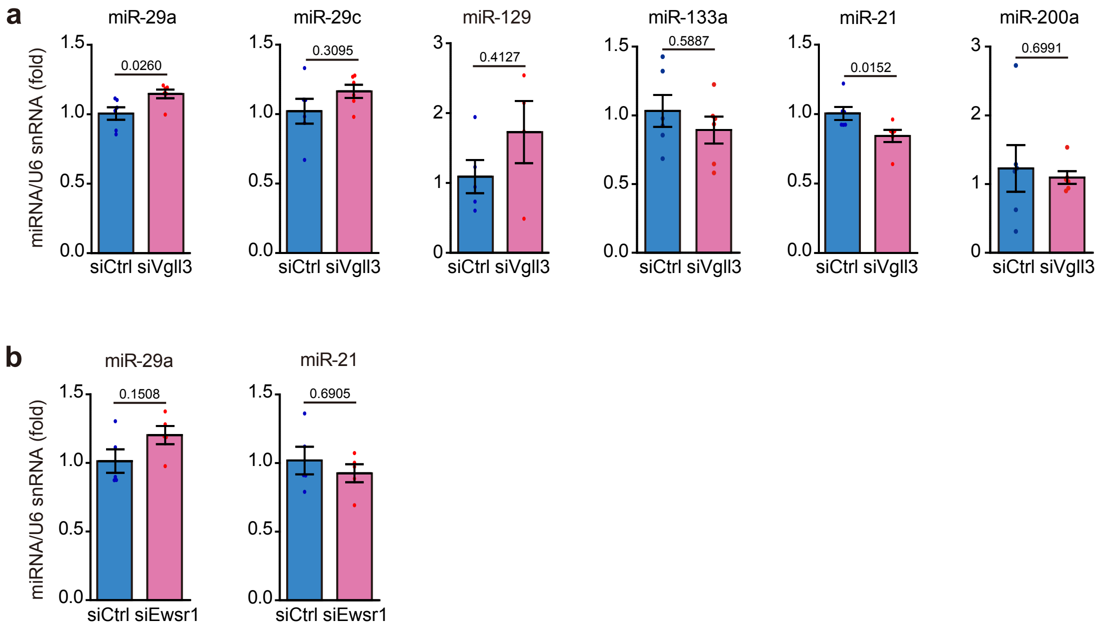

Supplementary Fig. 11 Expression levels of major miRNAs involved in fibrotic responses in cardiac myofibroblasts transfected with siRNA against *Vgll3* or *Ewsr1*.

a The expression level of miR-29a (*n* = 6 each), miR-29c (*n* = 6 each), miR-129 (siCtrl: *n* = 5, siVgll3: *n* = 4), miR-133a (*n* = 6 each), miR-21 (*n* = 6 each) and miR-200a (*n* = 6 each) in cardiac myofibroblasts transfected with siRNA targeting *Vgll3*. **b** The expression level of miR-29a and miR-21 in cardiac myofibroblasts transfected with siRNA targeting *Ewsr1* (*n* = 5 each). Data are shown as the mean ± SEM. *P* value was determined using two-sided Mann-Whitney's *U* test.

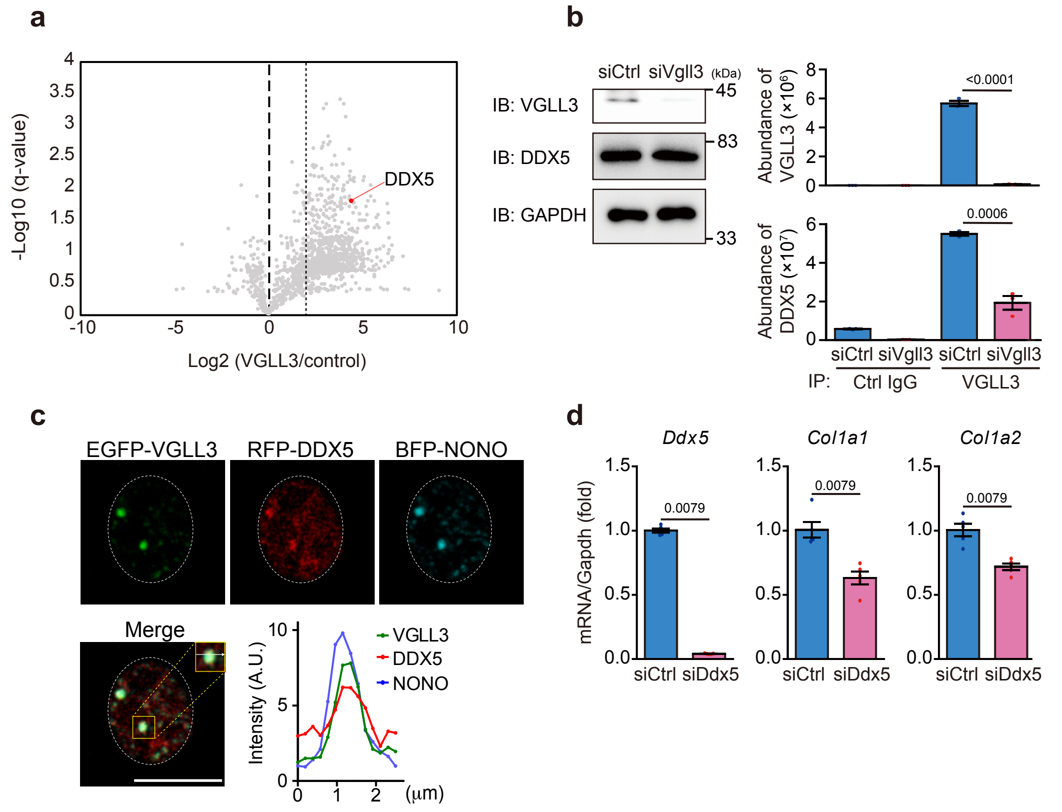

Supplementary Fig. 12 DDX5 interacts with VGLL3 and promotes the expression of collagens in cardiac myofibroblasts.

a DDX5 is indicated in volcano plot of Fig. 4**a**. **b** Endogenous interaction between VGLL3 and DDX5 in cardiac myofibroblasts analyzed by PRM mass spectrometry of immunoprecipitates with anti-VGLL3 antibody with three technical replicates. Western blot images of cell lysates used in the PRM analysis are shown in the left. **c** Images of live NIH3T3 cells transfected with EGFP-VGLL3, RFP-DDX5, and BFP-NONO. The graph represents line scans along the white line in the yellow dashed square in the inset on **c**. **d** mRNA levels of *Ddx5*, *Col1a1* and *Col1a2* in cardiac myofibroblasts transfected with siRNA targeting *Ddx5*. The mRNA levels were normalised to those of *Gadph* (n = 5 each). Results are shown as fold changes relative to siCtrl data. Data are presented as the mean ± SEM. P-values were determined using two-sided Student's t-test in **b** and two-sided Mann–Whitney's U test in **d**. Scale bars in **c** = 10 μm.

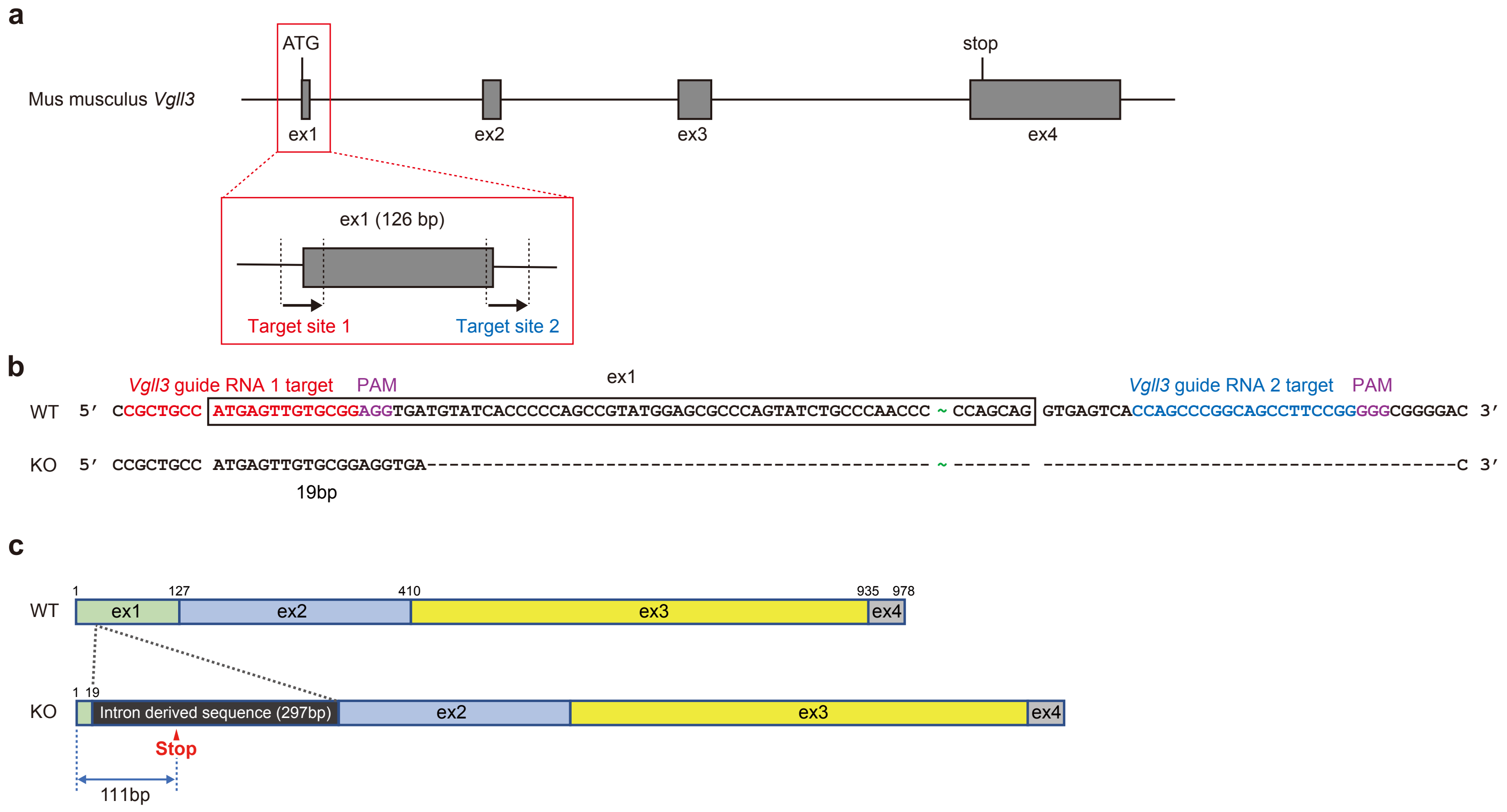

Supplementary Fig. 13 Generation of *Vgll3* KO mice.

a Schematic image of genomic target sites in the *Vgll3* gene. **b** Genomic sequences of the *Vgll3* locus in WT and *Vgll3* KO mice. Coloured characters indicate target sites 1 (red) and 2 (blue). Protospacer adjacent motifs (PAM) are labelled in purple. The green tilde (~) in ex1 indicates omission of the genome sequence. Figure dashes (-) indicate the missing genomic sequence in *Vgll3* KO mice. **c** A stop codon was newly generated in the *Vgll3* mRNA in *Vgll3* KO mice. In *Vgll3* mRNA from *Vgll3* KO mice, only the 1st–19th bases of exon 1 were included, and the intron-derived sequence was inserted between exon 1 and exon 2. Exon 1 deletion and intron insertion generated a stop codon (TAG) at the 37th amino acid, resulting in the incompletely functional VGLL3.

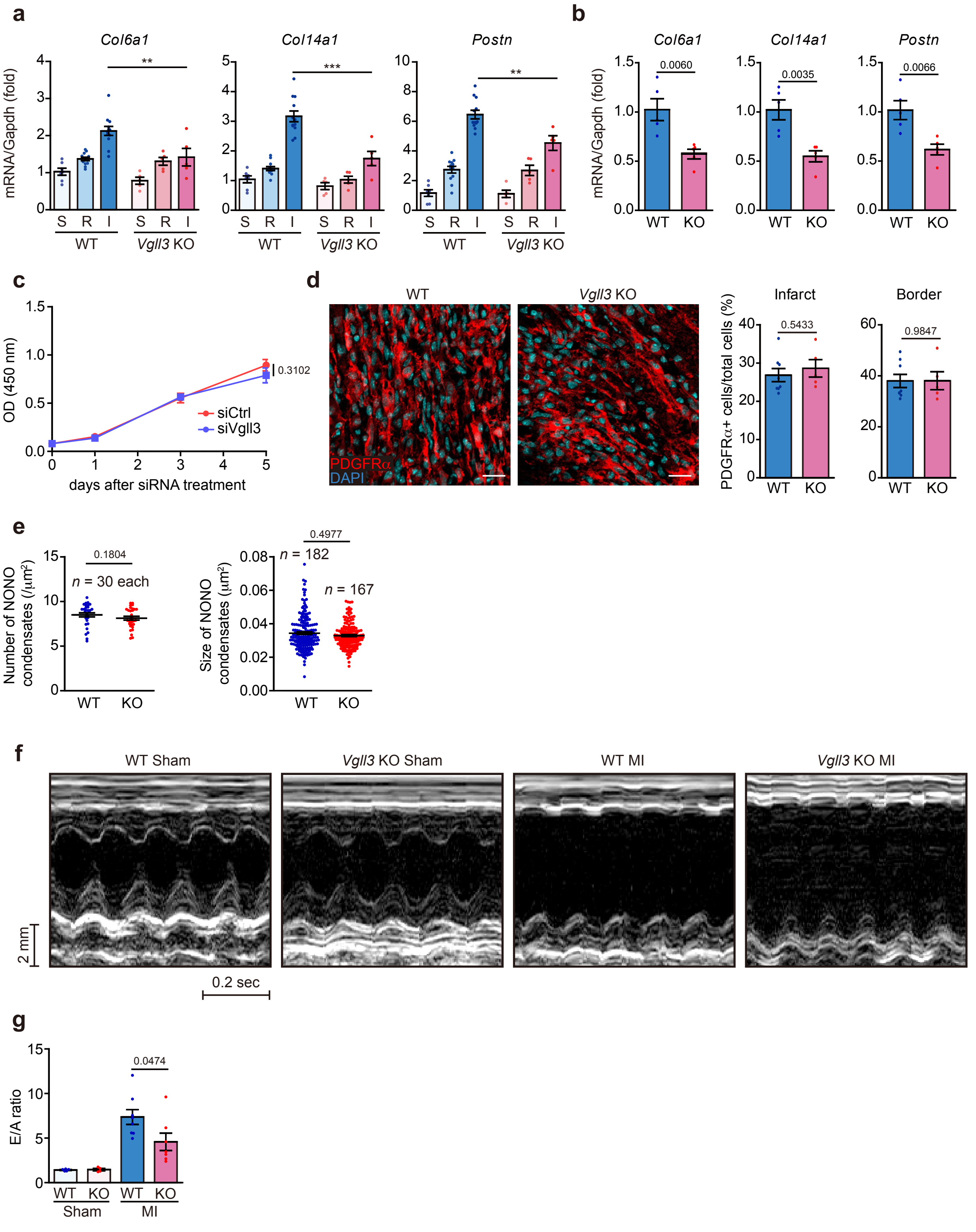

Supplementary Fig. 14 Cardiac conditions of WT and *Vgll3* KO mice after MI.
a mRNA levels of fibrosis-related genes in sham (S)-operated ventricles, and in the remote (R) and infarcted (I) areas of WT and *Vgll3* KO mouse hearts, 3 days after MI (WT: S/R/I, n = 7/12/12; KO: S/R/I, n = 5/5/5). **b** mRNA levels of fibrosis-related genes in cardiac myofibroblasts isolated from WT and *Vgll3* KO mouse hearts, 3 days after MI (WT: n = 5; KO: n = 5). **c** Cell proliferation of cardiac myofibroblasts transfected with siRNA targeting *Vgll3* was determined using WST-8 assay (n = 5). **d** Immunostaining images of PDGFRα+ fibroblasts in heart sections of WT and *Vgll3* KO mice, 4 days after MI. The percentages of PDGFRα+ fibroblasts in total cells in the infarcted or border area are shown in graph (n = 5). **e** The number (n = 30 cells, left) or the size (n > 150 condensates, right) of NONO condensates in cardiac myofibroblasts isolated from WT and *Vgll3* KO mouse hearts, 3 days after MI. **f** Representative M-mode images of WT and *Vgll3* KO mice, 28 days after MI or sham operation. **g** E/A ratio of WT and *Vgll3* KO mice, 28 days after MI or sham operation (WT: Sham/MI, n = 5/8; KO: Sham/MI, n = 4/7). Data in **a**-**e**, and **g** are presented as the mean ± SEM. *P*-values were determined using one-way ANOVA followed by Tukey's range test in **a**, two-sided Student's t-test in **b**-**d**, and **g**, and two-sided Mann–Whitney's *U* test in **e**, $^{**}P < 0.01$, $^{***}P < 0.001$. Scale bar in **d** = 20 μm.

Supplementary Table 1. Echocardiographic measurements and organ weights of WT and *Vgll3* KO mice on 28 days after MI.

HW; heart weight, BW; body weight. LW; lung weight, IVSTd; interventricular septal thickness diastolic, LVIDd; left ventricular inner diameter diastolic, LVPWd; left ventricular posterior wall diameter diastolic, LVIDs; left ventricular inner diameter systolic, %EF; percent ejection fraction, %FS; percent fractional shortening.

$^{\$}$ WT-MI vs WT-sham, $^{\#}$ KO-MI vs WT-MI, followed by two-sided Mann-Whitney's *U* test.

parameters	WT-sham (*n* =8)	WT-MI (*n* =18)	*Vgll3* KO-sham (*n* =5)	*Vgll3* KO-MI (*n* =7)
HW/BW (mg/g)	3.91±0.14	5.33±0.27$^{\$}$ (*P* =0.0004)	4.09±0.14	4.85±0.24
LW/BW (mg/g)	3.73±0.16	7.24±0.81$^{\$}$ (*P* =0.0003)	3.62±0.08	5.48±0.58
IVSTd (mm)	0.99±0.05	0.65±0.22$^{\$}$ (*P* =0.0003)	0.97±0.06	0.45±0.01
LVPWd (mm)	0.87±0.07	1.35±0.04$^{\$}$ (*P* =0.0002)	0.89±0.10	1.29±0.07
LVIDs (mm)	1.89±0.15	4.73±0.17$^{\$}$ (*P* <0.0001)	2.02±0.14	4.30±0.29
LVIDd (mm)	3.09±0.17	5.59±0.17$^{\$}$ (*P* <0.0001)	3.26±0.12	5.53±0.29
%EF	70.84±2.59	32.39±1.49$^{\$}$ (*P* <0.0001)	69.30±3.63	44.78±2.85$^{\#}$ (*P* =0.0022)
%FS	39.22±1.90	15.59±0.79$^{\$}$ (*P* <0.0001)	38.20±2.74	22.59±1.64$^{\#}$ (*P* =0.0012)

Supplementary Table 2. The rist of siRNAs used in this study

siRNA	Company	Assay ID	Species
siVgll3 #1	ambion	s91840	mouse
siVgll3 #2	ambion	s91839	mouse
siVGLL3	ambion	s52380	human
siNeat1	ambion	n256283	mouse
siEwsr1 #1	ambion	s65750	mouse
siEwsr1 #2	ambion	s125017	mouse
siDdx5	ambion	s64910	mouse
siVgll4	ambion	s107155	mouse
siTead1	ambion	s74928	mouse
siNono	ambion	s79235	mouse
siRbm14	ambion	s80246	mouse
siTardbp	ambion	s106687	mouse
siCtrl	ambion	4390844	mouse/human

Supplementary Table 3. List of plasmids used in this study

Transient expression

CDS	Vector	NCBI reference sequence	
HA-Vgll3	pcDNA3	NM_028572.2	NP_082848.1
EGFP-Vgll3	pcDNA3	CodonOptimization	NP_082848.1
EGFP-Vgll3 ΔIDR mutant	pcDNA3	CodonOptimization	
EGFP-Vgll3 GGS mutant	pcDNA3	CodonOptimization	
FLAG-Vgll1	pcDNA3	NM_001313763.1	NP_001300692.1
FLAG-Vgll2	pcDNA3	NM_153786.2	NP_722481.1
FLAG-Ewsr1	pcDNA3	NM_001283061.2	NP_001269990.1
RFP-Ewsr1	pcDNA3	NM_001283061.2	NP_001269990.1
BFP-NONO	pcDNA3	NM_023144.2	NP_075633.2

Retrovirus production

CDS	Vector	NCBI reference sequence	
FLAG-Vgll3	pMXs-puro	NM_028572.2	NP_082848.1
FLAG-Vgll3	pMXs-puro	CodonOptimization	NP_082848.1
FLAG-Vgll3 ΔIDR mutant	pMXs-puro	CodonOptimization	
FLAG-Vgll3 GGS mutant	pMXs-puro	CodonOptimization	
EGFP-Vgll3	pMXs-puro	CodonOptimization	NP_082848.1
HA-Vgll3	pMXs-puro	NM_028572.2	NP_082848.1
HA-Vgll4	pMXs-puro	NM_177683.3	NP_808351.1

Supplementary Table 4. List of antibodies and reagents used in this study

Western Blot

Antibody	Company name	Dilution	Catalogue number	Clone number
anti-VGLL3	Cosmobio	1:4000	custom-made	
anti-EWSR1	Protein tech	1:4000	Cat# 55191-1-AP	
anti-αSMA	Thermo	1:5000	Cat# MS-113-P	1A4
anti-Periostin	R&D systems	1:3000	Cat# AF2955	
anti-Collagen I	Abcam	1:10000	Cat# ab34710	
anti-Collagen 1a1	CST	1:5000	Cat#72026	E8F4L
anti-Rbm14	MBL	1:4000	Cat# RN069PW	
anti-TDP-43	MBL	1:4000	Cat# RN107PW	
anti-DDX5	BETHYL	1:3000	Cat# A300-523A	
anti-HA-HRP	Roche	1:10000	Cat# 12013819001	3F10
anti-FLAG-HRP	Sigma	1:20000	Cat# A8592	M2
anti-GAPDH	Santa Cruz	1:50000	Cat# sc-32233	6C5
anti-mouse IgG-HRP	Santa Cruz	1:10000	Cat# sc-2005	
anti-Rabbit IgG-HRP	Santa Cruz	1:10000	Cat# sc-2004	
anti-goat IgG-HRP	Santa Cruz	1:10000	Cat# sc-2768	
anti-rabbit IgG, HRP-linked Antibody	CST	1:2000	Cat# 7074	

Immunocytochemistry

Antibody	Company name	Dilution	Catalogue number	Clone number
anti-DYKDDDDK	Novus	1:200	Cat# NBP1-06712	L5
anti-VGLL3	Abcam	1:200	Cat# ab83555	
anti-YAP1	Sigma	1:200	Cat# WH0010413M1	2F12
anti-NONO	MBL	1:200	Cat# RN013MW	
anti-SFPQ	Proteintech	1:200	Cat# 15585-1-AP	
AlexaFluor®488-conjugated donkey anti-rabbit IgG	Invitrogen	1:200	Cat# A21206	
AlexaFluor®594-conjugated goat anti-rabbit IgG	Invitrogen	1:200	Cat# A11037	
AlexaFluor®488-conjugated donkey anti-rat IgG	Invitrogen	1:200	Cat# A11006	
AlexaFluor®546-conjugated goat anti-mouse IgG	Invitrogen	1:200	Cat# A11003	

Regent	Company name	Catalogue number
CellTrace Far Red	Thermo	Cat# C34572
DAPI	Dojindo	Cat# 340-07971
Latrunculin A	Abcam	Cat# ab144290
Y27632	Wako	Cat# 257-0051
BTT-3033	Tocris	Cat# 4724
VS-4718	Chemie Tek	Cat# CT-VS4718
Blebbistatin	Wako	Cat# 021-17041
Cell Permeable C3 Transferase	Cytoskeleton	Cat# CT04-A
1,6-Hexanediol	Sigma	Cat# 240117
2,5-Hexanediol	Sigma	Cat# H11904

Immunohistochemistry

Antibody	Company name	Dilution	Catalogue number	Clone number
anti-αSMA	Abcam	1:200	Cat# ab5694	
anti-CD45	BioLegend	1:200	Cat# 103101	30-F11
anti-alpha Actinin	Abcam	1:200	Cat# ab18061	
anti-PDGFRα	R&D systems	1:200	Cat# AF1062	
anti-Collagen 1a1	CST	1:200	Cat#72026	E8F4L
anti-human αSMA	Abcam	1:200	Cat# ab124964	
AlexaFluor®488-conjugated donkey anti-rabbit IgG	Invitrogen	1:200	Cat# A21206	
AlexaFluor®647-conjugated donkey anti-rabbit IgG	Jackson ImmunoResearch	1:200	Cat# 711-606-152	
AlexaFluor®488-conjugated donkey anti-rat IgG	Invitrogen	1:200	Cat# A11006	
AlexaFluor®488-conjugated donkey anti-mouse IgG	Invitrogen	1:200	Cat# A11001	
AlexaFluor®594-conjugated rabbit anti-goat IgG	Invitrogen	1:200	Cat# A 21223	

RNAscope

Gene	Species	Company name	Catalogue number
Negative probe	E.coli	ACD	Cat# 310034
Vgll3	mouse	ACD	Cat# 517821
Postn	mouse	ACD	Cat# 418581-C2
VGLL3	human	ACD	Cat# 515931
TSA Plus Cyanine 3		PerkinElmer	Cat# NEL753001KT
TSA Plus Cyanine 5		PerkinElmer	Cat# NEL745001KT

Stellaris RNA FISH

Gene	Species	Company name	Catalogue number
Neat1	mouse	LGC Biosearch Technologies	SMF-3010-1

Flow cytometry

Antibody	Company name	Dilution	Catalogue number	Clone number
PE-conjugated Rat anti-CD45 antibody	Biolegend	1:200	Cat# 103105	30-F11
FITC-conjugated Mouse anti-αSMA antibody	Sigma	1:200	Cat# F3777	1A4

Immunoprecipitation

Antibody and Beads	Company name	amount	Catalogue number	Clone number
nomal Rabbit IgG (0.4 mg/mL)	Santa Cruz	1 μg/sample	Cat# sc-2027	
anti-VGLL3 antibody (custom made) (0.21 mg/mL)	cosmo bio	1 μg/sample		
SureBeads Protein G	BioRad	5 μL/sample	Cat#1614023	
Pierce anti-HA Magnetic Beads	Thermo	20 μL/sample	Cat# 88837	2-2.2.14
anti-FLAG(DYKDDDDK) tag Antibody Magnetic Beads	Wako	20 μL/sample	Cat# 017-25151	1E6

Supplementary Table 5. List of qRT-PCR primer and probe sets used in this study

qRT-PCR

Gene Name	Species	Company name	Catalogue number	Forward	Rerverse	Probe
Vgll3	mouse	Sigma-Aldrich	Custom	5'-TGCCCTACAGCCTGCTATCA-3'	5'-CTCCTCCTCCTCCTCCTCTTG-3'	5'-CTTGCTGTATACCGCTAACTTCTGCTGGC-3'
Gapdh	mouse	Sigma-Aldrich	Custom	5'-CGTCCCGTAGACAAAATGGTGA-3'	5'-CCACTTTGCCACTGCAAATGG-3'	5'-CCAATACGGCCAAATCCGTTCACACCGA-3'
Collagen 1a1	mouse	Sigma-Aldrich	Custom	5'-CCCAAAGGTTCTCCTGGTGAAG-3'	5'-CGGTTTTGCCATCAGGACCA-3'	5'-TGGTGCCAAGGGTCTCACTGGCAGTC-3'
Collagen 1a2	mouse	Sigma-Aldrich	Custom	5'-CAAGCATGTCTGGTTAGGAGAGA-3'	5'-GCTGAGTTGCCATTTCCTTGG-3'	5'-CCCTTCTACGTTGTATTCAAACTGGCTGCC-3'
Collagen 3a1	mouse	Sigma-Aldrich	Custom	5'-AAGCCCTGATGGTTCTCGAAA-3'	5'-CTTGCAGCCTTGGTTAGGATC-3'	5'-ACCCAGTATTCTCCACTCTTGAGTTCGG-3'
Collagen 6a1	mouse	Sigma-Aldrich	Custom	5'-TTGCCCTGGTCATTACGGATG-3'	5'-TTGATTCCCACAGAAACTACCTGA-3'	5'-ACACTCAACGGGACACGACACCTCTCA-3'
Collagen 14a1	mouse	Sigma-Aldrich	Custom	5'-ATGGGCCAGTAAAGACAGATGC-3'	5'-GCAAGAGCAGGACCGTGGA-3'	5'-CAGGACTCTTCATCCCGCAGACCAGGAA-3'
Periostin	mouse	Sigma-Aldrich	Custom	5'-ACCTGCAATGACGAAGATCCAA-3'	5'-CAGGAACTCCATCTATGATTTTGGTG-3'	5'-TGGCTCTCCGTGGATCACTTCTGTCACC-3'
Neat1	mouse	Sigma-Aldrich	Custom	5'-TCGACCTCTGTCACTTTGCC-3'	5'-TGACAAGCTGCTCAGACCAG-3'	5'-CTTCCATGGTGCAGTGCATCTAGGCCT-3'
Ewsr1	mouse	Sigma-Aldrich	Custom	5'-CTACCCAATGCAGCCAGTCA-3'	5'-ACTGCTCTGATCGTAACTAGTCG-3'	5'-CGCACCTCCATCTTATCCTCCTACCAGCTA-3'
Cd68	mouse	Sigma-Aldrich	Custom	5'-CCGCTTATAGCCCAAGGAACA-3'	5'-TTCTGTGGCTGTAGGTGTCATC-3'	5'-AAAGGCCGTTACTCTCCTGCCATCCT-3'
Tnf	mouse	Sigma-Aldrich	Custom	5'-GTCTCAGCCTCTTCTCATTCCTG-3'	5'-GAGGCCATTTGGGAACTTCTCA-3'	5'-AGGGGCCACCACGCTCTTCTGTCTAC-3'
Vgll1	mouse	Sigma-Aldrich	Custom	5'-AAGAGGCCCCACAAATGCAG-3'	5'-TCTATCCGTGTGGCTGGTGT-3'	5'-TCCGTGTTTTATTCACCCCGTTCTTGAGGG-3'
Vgll2	mouse	Sigma-Aldrich	Custom	5'-GTCCCCCGCAGCCTTATTTC-3	5'-GCTGGGGTTGGGTTGGAAAA-3'	5'-CGCCTACACTCCCTACCACCAGAAACTAGC-3'
Vgll4	mouse	Sigma-Aldrich	Custom	5'-GTGAGCTTGCATGGTGGC-3'	5'-CACTGCTGTTCTTAGTCAGGG-3'	5'-CCTGTACGCATCTCTCCCCAGCCTCAT-3'
Acta2	mouse	Sigma-Aldrich	Custom	5'-CACCATGAAGATCAAGATCATTGCC-3'	5'-GGTAGACAGCGAAGCCAGGA-3'	5'-AGCCACCGATCCAGACAGAGTACTTGCG-3'
Oct4	mouse	Sigma-Aldrich	Custom	5'-TGAGGTGGAGTCTGGAGACC-3'	5'-GAACCATACTCGAACCACATCC-3'	5'-TGAAGTGCCCGAAGCCCTCCCTACA-3'
Thy1	mouse	Sigma-Aldrich	Custom	5'-ACCAGCCCTATATCAAGGTCCTTA-3'	5'-CACACTTGACCAGCTTGTCTCTA-3'	5'-AACTTCACCACCAAGGATGAGGGCGACT-3'
Tcf21	mouse	Sigma-Aldrich	Custom	5'-ACGACAAGTACGAGAACGGTTA-3'	5'-CCTTCAGGTCATTCTCTGGTTTG-3'	5'-CCACCATAAAGGGCCACGTCAGGTTGA-3'
Islr	mouse	Sigma-Aldrich	Custom	5'-GCCATCTCAAGAGTCTGGACC-3'	5'-GGCGCTGAGGCTGCTAAA-3'	5'-AGCCACAACCTCCTCTCTGAGTTTGCCT-3'
Nt5e	mouse	Sigma-Aldrich	Custom	5'-GATGGACAAACTTATCGCTCAGAAA-3'	5'-GGAAGGTGGATTTCCTGTGTAGA-3'	5'-TCCTCCCACCACGATGTCCACACCTC-3'
Vgll3 3'UTR	mouse	Sigma-Aldrich	Custom	5'-CAGCAGATCATATGGCAGCATG-3'	5'-TCTCCAGAAAAGGTTCAAATCGAAA-3'	5'-TGGCCTTCTATGTTCTCTTCTCACTCCACA-3'
Cyp7a1	mouse	Applied Biosystems	Assay ID: Mm00484150_m1			
Tead1	mouse	Sigma-Aldrich	Custom	5'-CAGAGAGACCCAGACTCGTACA-3'	5'-CAAGCCACCTTTCTTTTCAGGAAA-3'	5'-CATGCCAACCATTCTTACAGTGACCCGTT-3'
Nono	mouse	Sigma-Aldrich	Custom	5'-GGGAAAGGCATTGTTGAGTTCTC-3'	5'-CACAGGCCGAGGAAATGTAGTC-3'	5'-AGGAGCCTTCACTGCATCTGTCCAGAGC-3'
Rbm14	mouse	Sigma-Aldrich	Custom	5'-GCCCCTGAACACTTGGAAGAT-3'	5'-ATGTGAACAAACGCGTAGTCTTT-3'	5'-ACATGCAGCCGATACATTGCCCACGA-3'
Tardbp	mouse	Sigma-Aldrich	Custom	5'-TGGGCGATGGTGTGACTGTA-3'	5'-CGTCCAACAAACACCTTTCTGC-3'	5'-ACTTCCCAACTCTAAGCAAAGCCCAGACGA-3'
Ddx5	mouse	Sigma-Aldrich	Custom	5'-CGAAGACAGAGGTTCAGGTCG-3'	5'-CACCCCTTTTGCCTGCAGAG-3'	5'-CGTCCTTCATGCCTCCTCTACCCCTGG-3'
ACTA2	human	Sigma-Aldrich	Custom	5'-GATCCTGACTGAGCGTGGC-3'	5'-CAGAGCTACATAACACAGTTTCTCC-3'	5'-ATTCCTTCGTTACTACTGCTGAGCGTGAGA-3'
Collagen 1a1	human	Sigma-Aldrich	Custom	5'-ACTGGTGACCTGCGTGTA-3'	5'-GCCGCATACTCGAACTGG-3'	5'-CTCTCGCCAACCAGACATGCCTCTTG-3'
Collagen 1a2	human	Sigma-Aldrich	Custom	5'-CTGGCTTAAAGGGACACAATGGA-3'	5'-CCAGCAGGACCCACGGAG-3'	5'-CAAGGTCTGCCTGGTATCGCTGGTCAC-3'
VGLL3	human	Sigma-Aldrich	Custom	5'-ACCACCCTCATGCCCACA-3'	5'-CACACTGGGGAGCAGGAATC-3'	5'-CCACCGCCACCATCATCACCATCACCA-3'
GAPDH	human	Sigma-Aldrich	Custom	5'-TGGGCTACACTGAGCACCAG-3'	5'-GCCAAATTCGTTGTCATACCAGG-3'	5'-TTCAACAGCGACACCCACTCCTCCACC-3'
18S rRNA	mouse/human	Sigma-Aldrich	Custom	5'-GGGTCATAAGCTTGCGTTGATTAAG-3'	5'-TCCGAGGGCCTCACTAAACC-3'	5'-TACACACCGCCCGTCGCTACTACCG-3'
miR-21	mouse/human	Applied Biosystems	Assay ID: 000397			
miR-29a	mouse/human	Applied Biosystems	Assay ID: 002112			
miR-29b	mouse/human	Applied Biosystems	Assay ID: 000413			
miR-29c	mouse/human	Applied Biosystems	Assay ID: 000587			
miR-129	mouse/human	Applied Biosystems	Assay ID: 000590			
miR-133a	mouse/human	Applied Biosystems	Assay ID: 002246			
miR-200a	mouse/human	Applied Biosystems	Assay ID: 000502			
U6 snRNA	mouse/human	Applied Biosystems	Assay ID: 001973			

Supplementary Table 6. List of primers used in this study

PCR

Gene Name	Species	Company name	Forward	Rerverse
Vgll1	mouse	eurofins	5'-CTCTTCTTGGACAATTGCACAGCCAGCAGC-3'	5'-CTTTGGAGATGGGCTTCTTTCATCATCAAG-3'
Vgll2	mouse	eurofins	5'-CAAAGCACACAGAAGCTCTGGACCCTGGAG-3'	5'-CTGCATTCCCTCCTCCGCTTACCTGAGTCC-3'
Vgll3	mouse	eurofins	5'-GACATTGGGTCAGTAGTGGATGAACACTTC-3'	5'-GACTGTAGTCAGATCTGTCTTGGTGATGTC-3'
Vgll4	mouse	eurofins	5'-GAATAAGACTGTCAACGGAGACTGCCGCAG-3'	5'-CAGGAGACCACAGAGGGGGAGTGACTATGG-3'

Supplementary Figure 5

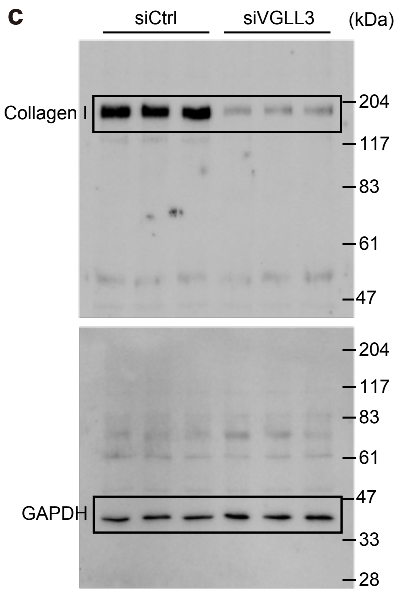

Supplementary Figure 12

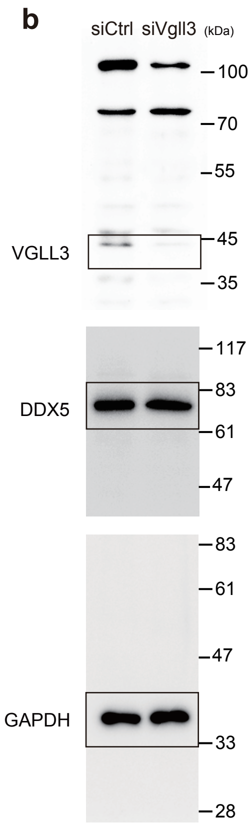

Supplement: Supplementary file 1 — Supplementary Information [file 41467_2023_36189_MOESM1_ESM.pdf]
